# Base excision repair accessory factors in senescence avoidance and resistance to treatments

**DOI:** 10.20517/cdr.2022.36

**Published:** 2022-06-22

**Authors:** Elise Vickridge, Camila C. F. Faraco, Alain Nepveu

**Affiliations:** ^1^Goodman Cancer Institute, McGill University, 1160 Pine avenue West, Montreal, Québec H3A 1A3, Canada.; ^2^Departments of Biochemistry, McGill University, 1160 Pine avenue West, Montreal, Québec H3A 1A3, Canada.; ^3^Medicine, McGill University, 1160 Pine avenue West, Montreal, Québec H3A 1A3, Canada.; ^4^Oncology, McGill University, 1160 Pine avenue West, Montreal, Québec H3A 1A3, Canada.; ^#^These authors contributed equally to this work.

**Keywords:** Base excision repair, reactive oxygen species, DNA repair accessory factor, oxidative DNA damage, resistance to treatment, tumour heterogeneity, acquisition of resistance

## Abstract

Cancer cells, in which the RAS and PI3K pathways are activated, produce high levels of reactive oxygen species (ROS), which cause oxidative DNA damage and ultimately cellular senescence. This process has been documented in tissue culture, mouse models, and human pre-cancerous lesions. In this context, cellular senescence functions as a tumour suppressor mechanism. Some rare cancer cells, however, manage to adapt to avoid senescence and continue to proliferate. One well-documented mode of adaptation involves increased production of antioxidants often associated with inactivation of the *KEAP1* tumour suppressor gene and the resulting upregulation of the NRF2 transcription factor. In this review, we detail an alternative mode of adaptation to oxidative DNA damage induced by ROS: the increased activity of the base excision repair (BER) pathway, achieved through the enhanced expression of BER enzymes and DNA repair accessory factors. These proteins, exemplified here by the CUT domain proteins CUX1, CUX2, and SATB1, stimulate the activity of BER enzymes. The ensued accelerated repair of oxidative DNA damage enables cancer cells to avoid senescence despite high ROS levels. As a by-product of this adaptation, these cancer cells exhibit increased resistance to genotoxic treatments including ionizing radiation, temozolomide, and cisplatin. Moreover, considering the intrinsic error rate associated with DNA repair and translesion synthesis, the elevated number of oxidative DNA lesions caused by high ROS leads to the accumulation of mutations in the cancer cell population, thereby contributing to tumour heterogeneity and eventually to the acquisition of resistance, a major obstacle to clinical treatment.

## BASE EXCISION REPAIR

### Overview

**Figure 1:**
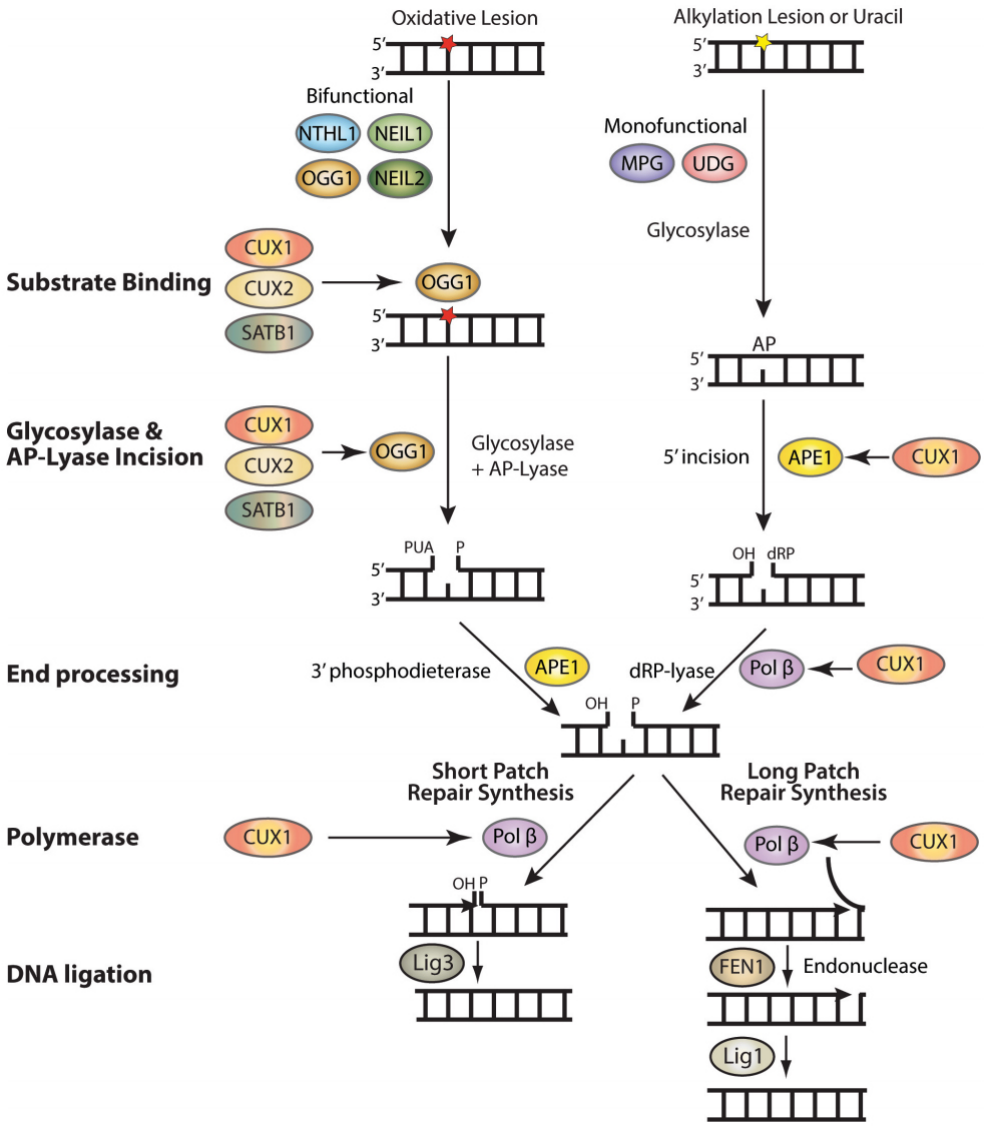
Auxiliary factors stimulate the enzymatic activities of several BER enzymes. BER is initiated by the removal of a damaged base by a glycosylase which creates an apyrimidinic or apurinic site (AP site). Bifunctional glycosylases such as NTHL1, OGG1, NEIL1, and NEIL2 have an AP-lyase activity and can introduce a single-strand break. APE1 then removes the phosphor-α,β-unsaturated aldehyde (PUA) to create a single-strand break with a 3’-OH and a 5’-phosphate. In the case of MPG and UDG, both monofunctional glycosylases, APE1 will cleave the backbone, leaving a single-strand break with a 3’dRP that will be removed by Pol β. In short patch repair, Pol β inserts the missing nucleotide and Lig3 seals the break. In long patch repair, Pol β inserts multiple nucleotides, FEN1 cleaves the displaced strand, and Lig1 seals the break. The CUT domain proteins CUX1, CUX2, and SATB1 stimulate the binding of OGG1 to 8-oxo-deoxyguanine as well as its glycosylase and AP-lyase enzymatic activities. The CUT domains of CUX1 have also been shown to stimulate the 5’-incision activity of APE1 as well as the dRP-lyase, DNA polymerase, and strand-displacement activities of DNA Pol β.

Base excision repair (BER) repairs most base lesions including alkylated, deaminated and oxidized bases, as well as apurinic/apyrimidinic (AP) sites [Figure 1]^[1]^. This pathway is initiated by one of many DNA glycosylases, which recognizes a specific base lesion and cleaves the N-glycosylic bond linking the altered base to the DNA backbone to produce an apyrimidinic/apurinic site (AP site )^[1,2]^. In mammals, AP sites are targeted by the AP endonuclease 1, APE1, which incises the DNA backbone 5’ to the AP site to generate a single-strand break with a 5’-deoxyribose phosphate (dRP)^[3]^. In addition, DNA glycosylases for oxidized bases are also endowed with an AP/lyase activity that generates a single-strand nick 3’ to the AP site. OGG1 and NTHL1 generate the single-strand nick through beta elimination, while NEIL1 and NEIL2 do this by beta delta elimination (reviewed in^[4]^). 5’ or 3’ end processing of the resulting single-strand breaks is then performed by DNA Pol β, APE1, or PNKP, and repair synthesis and ligation are accomplished by the short-patch or long-patch pathways^[5-7]^. In short-patch repair, DNA Pol β (Pol β) adds a single base and removes the 5’- dRP to allow ligation^[8]^. In long patch repair, 2 to 13 bases are synthesized by Pol β or δ/ε, thereby generating a displaced strand that is cleaved by the flap structure-specific endonuclease FEN1 prior to ligation^[1,9]^.

The importance of BER is illustrated by its complex implications in cancer. On the one hand, inherited as well as somatic mutations in some BER genes were found in various cancers and in some cases have been linked to particular mutation signatures. On the other hand, overexpression of BER enzymes has been reported in many tumours and cancer cell lines and has been associated with increased resistance to genotoxic treatments.

### Germ line and somatic mutations in BER genes that predispose to cancer

The role of Pol β in maintaining genome integrity is illustrated by the fact that mouse embryo fibroblasts from Pol β-null mice exhibit a 1.6-fold increase in spontaneous mutation frequency^[10]^. However, it is important to consider that the role of Pol β in the repair of endogenous DNA lesions does contribute to the acquisition of mutations. The average Pol β error rate was estimated at 7 × 10^-4^ for 12 possible base substitution errors and at 3 to 9 × 10^-4^ for single base deletions^[11]^. In a diploid mammalian cell, there are approximately 30,000 endogenous DNA base lesions per day^[12]^ (and table 2.1 in^[13]^). The term “endogenous” here means that these lesions are caused by normal metabolism in contrast to exogenous sources of DNA damage. Considering a Pol β error rate of at least 1/1000, we can estimate that 30 mutations per cell are acquired every day as a result of BER activity. This said, inherited and somatic mutations that alter the function of BER enzymes increase the risk of cancer, and for this reason, BER is considered to function as a tumour suppressor mechanism.

Approximately 30% of tumours analyzed exhibit a mutation in the *Pol β* gene, and there is evidence that these Pol β variants can contribute to tumour development^[14]^. The Pol β^K289M^ variant was found to cause a 2.5-fold increase in mutation frequency^[15]^. Moreover, stable expression of this Pol β^K289M^ variant, as well as another variant, Pol β^I260M^, was found to induce a transformed phenotype in mouse cells, as judged from focus formation on a monolayer and a soft-agar assay^[16]^.

In addition to somatic mutations, some germline mutations in BER proteins predispose to cancer. MUTYH-associated polyposis (MAP) was first described when two inherited germline mutations in the *MUTYH *gene (Y165C and G382D) were identified in three siblings with clinical symptoms of familial adenomatous polyposis (FAP), an inherited condition that predisposes to colorectal cancer (CRC)^[17]^. *MUTYH* is the human homologue of MutY, a DNA glycosylase that removes adenine from 8-oxoG:A base pairs, preventing G:C to T:A transversions (reviewed in^[18]^). The adenine glycosylases encoded by the *MUTYH^Y165C^* and *MUTYH^G382D^* mutants displayed much reduced activity^[17]^. Further investigations confirmed that inherited *MUTYH* mutations increase the risk of colorectal polyposis as well as other cancers including duodenal carcinoma, bladder, ovarian, and skin cancer^[19-23]^.

More recently, a homozygous nonsense mutation in the *NTHL1* DNA glycosylase gene was also identified in individuals with multiple colonic adenomas from three unrelated families, suggesting a new BER-associated predisposing condition for colorectal cancer^[24]^. This mutation confers a highly penetrant predisposition to adenomatous polyposis and is also associated with other benign and malignant lesions^[25]^. The NTHL1-associated polyposis (NAP) and findings of NTHL1 variants and altered regulation in tumors, provide another evidence on the role of BER in maintaining genomic stability and preventing cell transformation^[26]^. Note that the patterns of somatic mutations associated with MAP and NAP are distinct and are related to specific mutation signatures^[25,27,28]^.

## COOPERATING EVENTS IN TUMOUR DEVELOPMENT

The notion of multi-step carcinogenesis arose from early experiments with chemical carcinogens that were classified as initiators and promoters^[29]^. Following the identification of cellular oncogenes, experiments in tissue culture revealed that activation of a single oncogene is not sufficient for cell transformation and tumour development^[30]^. Indeed, while the introduction of RAS oncogenes into immortalized rodent cells was able to produce transformed cell foci on a monolayer or in soft agar^[31]^, the same procedure was ineffective when applied to primary cells^[30]^. Soon the concept of cooperation between oncogenes emerged when it was shown that the MYC and RAS oncogenes are able to produce transformed cell foci when co-expressed in primary rat fibroblasts^[30]^. The family of RAS proteins comprises three homologous proteins, HRAS, KRAS, and NRAS. RAS proteins serve as transducers of cell surface receptors to intracellular effector pathways. They alternate between an inactive GDP-bound state and an active GTP-bound state. Most mutations occurring in a RAS oncogene will impair its GTP hydrolysis function such that RAS remains in a GTP-bound active state. This leads to sustained activation of downstream effector pathways such as proliferation and differentiation^[32,33]^. In addition to missense mutations, there is evidence suggesting that RAS mutant overexpression is required for tumour development^[34]^. Indeed, it was proposed that Ras-induced tumorigenesis involves two steps: the acquisition of an activating mutation and overexpression of the activated Ras allele^[34]^.

The cooperation between MYC and RAS received additional confirmation from experiments in transgenic mice: mice that expressed both MYC and RAS transgenes developed more tumours and with a shorter latency period^[35]^. The concept of cooperating events was extended with the realization that a RAS oncogene could also collaborate with a dominant-negative mutant of p53 or a viral protein such as Large T or Adenovirus E1A that inactivates a tumour suppressor protein^[36-39]^. Not only can two oncogenes cooperate, but an oncogene can also cooperate with the inactivation of a tumour suppressor gene. A further conceptual advance in our understanding of cooperating events was realized with the description of stress phenotypes of cancer cells^[40]^. In essence, the tumorigenic state generates additional pressures that impose a block on cell proliferation and eventually induce cellular senescence or apoptosis. To surmount this block and continue to proliferate, cancer cells require extensive adaptation in cellular processes that are not oncogenic per se. As a result, cancer cells become acutely dependent on the increased activity of some normal proteins. The term “oncogene-addiction” had previously been introduced to illustrate the dependence of cancer cells on a tyrosine kinase oncogene such as BCR-ABL^[41,42]^. The dependence of cancer cells on non-oncogenic proteins was designated “non-oncogene addiction”^[40]^. In summary, tumour development involves cooperation between the activation of some oncogenes, the inactivation of some tumour suppressors and the increased expression and/or activity of several normal proteins. A striking case of extensive adaptation is illustrated by the response of cancer cells to oxidative stress and oxidative DNA damage.

## OXIDATIVE STRESS IN CANCER CELLS AND CELLULAR SENESCENCE

**Figure 2:**
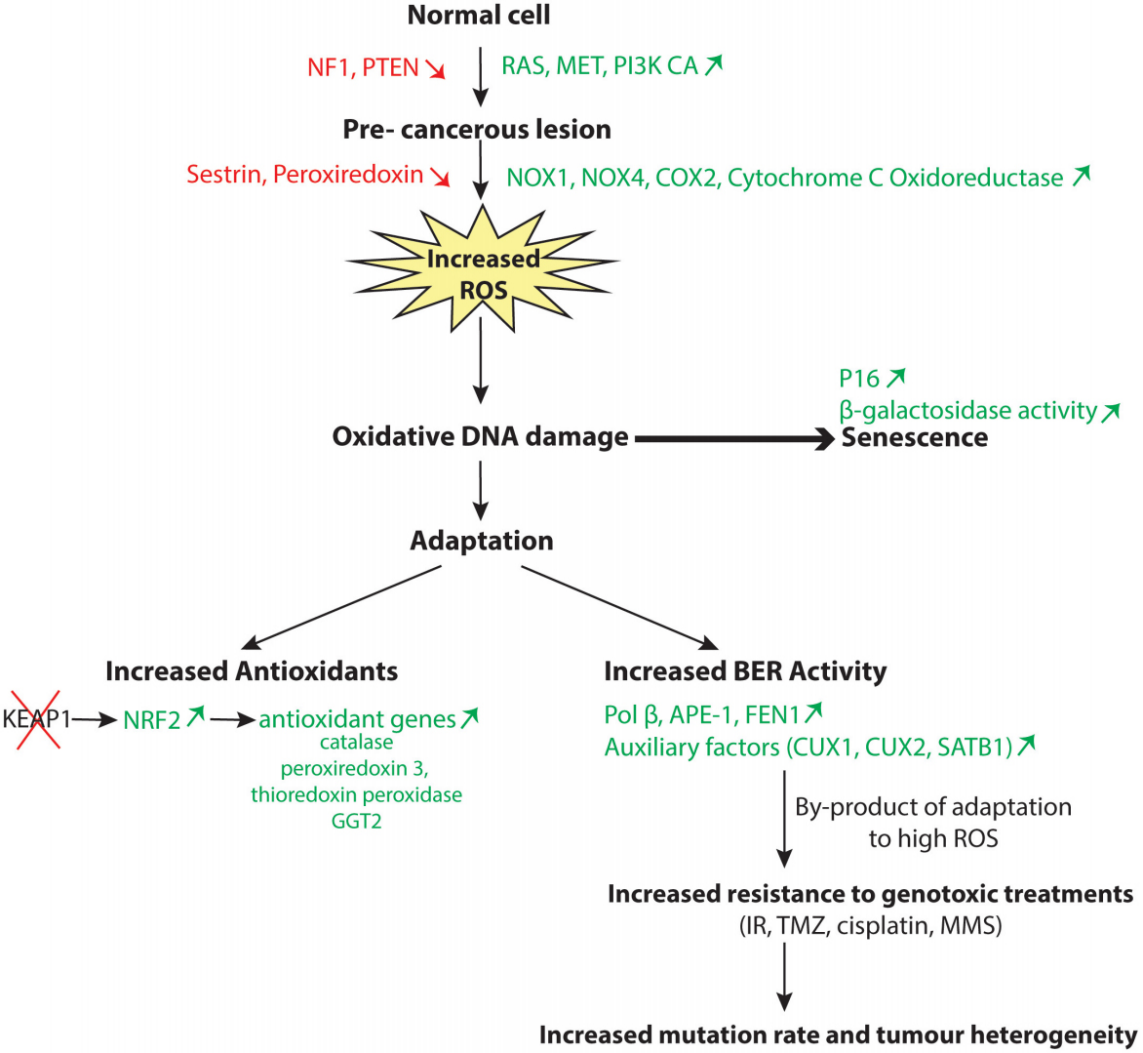
Adaptation of cancer cells to oxidative stress. Activation of the RAS or PI3K pathway leads to elevated production of reactive oxygen species (ROS), which causes oxidative DNA damage and, ultimately, cellular senescence. Two modes of adaptation have been described to enable cancer cells to avoid cellular senescence and continue to proliferate. Some cancer cells increase their antioxidant capabilities notably following genetic inactivation of the Kelch-like ECH-associated protein 1 (*KEAP1)* gene which leads to nuclear factor erythroid-2 related factor 2 (NRF2) upregulation and the subsequent transcriptional activation of antioxidant genes. Alternatively, some cancer cells increase their BER capacities by overexpressing BER enzymes such as Pol β, APE1, and FEN1 or auxiliary factors such as CUX1, CUX2, or SATB1. As a by-product of increased BER activity, these cancer cells exhibit resistance to genotoxic treatments like radiation therapy(IR), temozolomide (TMZ), cisplatin, or methyl methanesulfonate (MMS).

Among the stress phenotypes of cancer cells, oxidative stress and resulting oxidative DNA damage have emerged as critical players. The study of mouse fibroblasts transformed with a RAS oncogene revealed that these cells produce large amounts of reactive oxygen species (ROS)^[43]^. In fact, elevated ROS production is not limited to cancer cells that harbour a RAS oncogene but has also been observed in cancer cells that harbour a mutation in another gene of the RAS or the phosphatidylinositol-3 kinase (PI3K) pathway (NF1, PIK3CA, PTEN) or an upstream tyrosine kinase (Met or BCR-ABL)^[44-51]^. Mechanistic studies identified multiple sources of ROS production in cancer cells, such as increased expression and activity of the enzymes NADPH oxidase 1 and 4 (NOX1, NOX4) and cyclooxygenase-2 (Cox-2), transcriptional repression of sestrin family genes, and increased ROS production by the cytochrome c-oxidoreductase in the mitochondrial electron transport chain^[52-59] ^[Figure 2]. Sestrins play an important role in the regeneration of peroxiredoxins and, as such, contribute to the antioxidant firewall^[60]^. Importantly, as cell proliferation was inhibited by treatment with a chemical antioxidant or knockdown of genes encoding these respective enzymes, it was concluded that ROS play an important role in mediating the mitogenic effect of RAS^[43,52,54-57]^. However, it was quickly realized that following an initial period of accelerated proliferation, elevated ROS levels also cause the appearance of cells that exhibit markers of cellular senescence such as p16^INK4a^ expression and β-galactosidase activity at pH 6^[61]^. The induction of cellular senescence by oncogenes was rapidly confirmed in mouse models^[62,63]^. Experiments in the animal also introduced an important twist to the unfolding story. Indeed, senescent cells were detected in premalignant tumours but not in malignant ones^[62]^. For example, BRAF^V600E ^induced benign lung tumours that only rarely progressed to adenocarcinoma^[63]^. Likewise, in human tissues, senescent cells were not detected in malignant tumours but were observed in pre-cancerous lesions such as colon adenomas^[64-66]^, benign tumours of melanocytes (naevi) caused by the *BRAF^V600E^* mutation^[67]^, and neurofibromas resulting from Neurofibromatosis (NF1) inactivation^[68]^. In this context, cellular senescence is deemed a tumour suppression mechanism that functions by eliminating early neoplastic cells from the proliferative pool (reviewed in^[69]^). It should be noted, however, that the presence of senescent cells in tissues has the potential to stimulate the proliferation of tumour cells through the senescence-associated secretory pathway (reviewed in^[69]^).

## ADAPTATION OF CANCER CELLS TO OXIDATIVE STRESS

Cellular senescence protects us against tumour development by preventing the proliferation of cancer cells that exhibit higher ROS levels. Unfortunately, some rare cancer cells manage to adapt by developing mechanisms to prevent the deleterious effects of excessive ROS levels [Figure 2].

### Increased production of antioxidants

One mechanism of adaptation involves the increased production of antioxidants [Figure 2]. Early studies using a proteomic approach documented the upregulation of enzymes involved in cellular redox balance, including catalase, peroxiredoxin 3, thioredoxin peroxidase, and γ-glutamyltransferase 2 (GGT2)^[70-72]^. Subsequent studies described various mechanisms by which cancer cells maintain the ratio of reduced to oxidized glutathione (GSH/GSSG) and nicotinamide adenine dinucleotide phosphate (NADPH/NADP+), notably by reprogramming the metabolism of glutamine, glucose, and fatty-acid^[73-76]^. Some important players in the response of cancer cells to elevated ROS levels are the nuclear factor erythroid-2 related factor 2 (NRF2) and its repressor protein Kelch-like ECH-associated protein 1 (KEAP1) [Figure 2]. NRF2 is a redox-sensitive transcription factor that binds to the antioxidant response element (ARE) and activates many genes that code for antioxidants and detoxification proteins^[77]^. KEAP1 binds to NRF2 and negatively regulates its activity by targeting it to proteasomal degradation^[78]^. In normal cells, the interaction between KEAP1 and NRF2 is disrupted by oxidants and electrophilic agents, thereby allowing NRF2 to translocate to the nucleus and activate the transcription of antioxidant genes^[78]^. In many cancer cells, NRF2 has become constitutively active following the acquisition of inactivating mutations within the *KEAP1* gene or missense mutations within either the *KEAP1* or *NRF2* gene that weaken the interaction between the two proteins^[79-83] ^(reviewed in^[84]^). It was estimated that somatic mutations in KEAP1 and NRF2 are present in 15% and 10% of lung cancers, respectively^[84]^. The existence of mutations that disrupt the KEAP1/NRF2 regulatory system in cancer cells sheds new light on the observed “oncogene-directed” increased expression of NRF2 whereby the expression of KRAS, BRAF, and MYC oncogenes in primary murine cells was found to cause an increase in NRF2 mRNA and protein levels^[85]^. This and other studies suggested a causal link between RAS and increased NRF2 expression and its transcriptional targets as if a downstream effector of the RAS pathway would act directly to upregulate NRF2^[58,70-72,76,85]^. Instead of direct induction of NRF2 by the RAS pathway, we envision that cancer cells with higher expression of antioxidants emerge by a Darwinian process of natural selection. Following sustained activation of the RAS pathway, virtually all cells in a population will be negatively affected by excessive ROS levels, but rare cells that express high levels of antioxidants will continue to proliferate and will gradually represent an increasing fraction of the population. This notion is supported by two observations. First, ectopic expression of KRAS or other oncogenes in murine primary cells did not have any effect on NRF2 expression when investigated one week after the retroviral infection^[85]^. Increased NRF2 expression was observed only in mouse embryo fibroblasts derived from mice that harbour the KRAS oncogene or another oncogene^[85]^. Therefore, we suppose that embryonic development up to day 13 allowed enough time for some adaptation. Secondly, if the RAS pathway was able to directly upregulate NRF2 expression, tumour development would not require somatic mutations in *KEAP1* or *NRF2* that abrogate the interaction between the two proteins.

### Increased capacity to repair oxidative DNA damage

Several observations concur to indicate that adaptation to elevated ROS production in cancer cells can involve an increase in DNA repair capacity, notably of the BER pathway [Figure 2].

#### Overexpression of BER enzymes in cancer

Early studies have documented the increased expression and/or activity of BER enzymes in tumour samples. Pol β was first reported to be overexpressed in many cancer cell lines as well as in breast, colon, and prostate adenocarcinomas^[86]^. The analysis of 68 tumour samples of various tissue of origin estimated that Pol β was overexpressed between 2 to 12-fold in 40% of samples^[87]^. Pol β expression and activity were significantly higher in blood samples from 9 chronic myelogenous leukemia patients than that from healthy donors^[88]^.

Immunohistochemistry analysis of a panel of ovarian cancers revealed a wide range of APE1 expression with considerable heterogeneity within the same tumour^[89]^. In prostate tissue, APE1 overexpression correlates with tumour progression: it was detected in 6 out of 100 (6%) benign prostate hypertrophy, 58 out of 100 prostatic intraepithelial neoplasia, and 67 out of 100 (67%) prostate cancers^[90]^. Measurements of APE1 activity in extracts of 58 glioblastomas and adjacent histologically normal brains revealed increased enzyme activity in 93% of tumour/normal pairs^[91]^. APE1 activity was also greater in high-grade than in low-grade tumours^[91]^. APE1 was found to be overexpressed in 43 out of 60 (72%) osteosarcomas, and a significant correlation was observed between elevated APE1 expression and shorter survival^[92]^. The analysis of 82 cases of oral tongue squamous cell carcinoma showed higher APE1 expression in 53 (64%) samples^[93]^.

The flap structure-specific endonuclease (FEN1) has been reported to be highly expressed in lung cancer cell lines and in multiple types of tumours, including neuroblastomas, prostate, gastric, pancreatic, and non-small cell lung cancers^[94-98]^.

#### BER enzymes identified in a synthetic lethality screen of RAS-driven cancer cells

A requirement for an efficient BER pathway in RAS-driven cancer cells was first suggested from a genome-wide screen to identify synthetic lethal interactions with the RAS oncogene^[99]^. The screen was performed using a pair of isogenic colorectal cell lines (DLD-1) that carry or not an endogenous activating KRAS^G13D^ mutation. While stringent statistical criteria (*P* ≤ 0.1) identified 368 genes whose knockdown is synthetic lethal to KRAS^G13D^-driven cancer cells, a more relaxed cut off (*P* ≤ 0.3) extended this list to 1741 genes, among which were four genes involved in BER: *NEIL2*, *XRCC1*, *POLβ*, *LIG3*^[99]^. These results indicated that these four BER genes are required for the proliferation of KRAS^G13D^-driven DLD-1 cells to a greater extent than for the control cells. The *CUX1* gene was also found in the list of potential synthetic lethality genes, but the molecular basis for its requirement was not understood until later.

#### Requirement for CUX1 function as a BER accessory factor in RAS-driven cancer cells

Transgenic mice expressing the p200 CUX1 protein under the control of the mouse mammary tumour virus regulatory sequences developed mammary tumours with a penetrance of ~40% but with a long latency period of 70 to 100 weeks^[100]^. Such a long latency period indicates that *CUX1* is probably not an oncogene per se, and that, certainly, other cooperating events are required for tumour development in cells that overexpress CUX1. Interestingly, 44% of tumours from *Cux1* transgenic mice harboured an activating mutation at codon 12 or 61 of *Kras*. Such a high frequency of spontaneous mutations is striking. This implies that the probability that a mouse will acquire an activating mutation in the *Kras* oncogene during its life is approximately 17% (40% × 44%). Yet, wild-type mice do not develop cancer at this rate. The reason for this, fortunately, is that RAS oncogenes trigger a cellular senescence response after an initial period of accelerated proliferation^[62-68]^ (reviewed in^[69]^). Therefore, other cooperating events are required for pre-cancerous cancer cells to avoid senescence and continue to proliferate. The cooperation of CUX1 with a RAS oncogene in tumour development was confirmed by performing lentiviral infections in the lung of mice. The combination of CUX1 with RAS produced a higher number of cancerous lesions of a larger size than RAS alone. Importantly, while RAS alone caused the appearance of adenomas that exhibited hallmarks of cellular senescence, the combination of CUX1 and RAS produced higher grade adenomas which, in one case, evolved to the adenocarcinoma stage^[100]^. DNA repair assays *in vitro* with purified proteins established that the p200 CUX1 protein functions as an accessory protein that stimulates the enzymatic activities of several BER enzymes: OGG1, APE1, and Pol β [Figure 1]^[100-102]^. Overexpression of p200 CUX1 accelerates the repair of oxidative DNA damage following treatment with H_2_O_2,_ whereas CUX1 knockdown increases genomic DNA damage, as observed by comet assays and the measurement of abasic sites and 8-oxo-deoxyguanine bases^[100]^. While the introduction of a RAS oncogene into cells causes an increase in ROS levels that are associated with an increase in DNA damage and the number of senescent cells, simultaneous expression with p200 CUX1 eliminates the increase in genomic DNA damage and senescent cells without affecting ROS elevation^[100]^. In normal cells, the role of CUX1 as a BER accessory factor is not essential for survival, as demonstrated from lethality screens in human cells and the viability of *Cux1^-/-^* knockout mice^[103-106]^. This biochemical activity of CUX1 appears to be needed only in abnormal situations of oxidative stress. For example, mouse embryo fibroblasts (MEFs) from *Cux1^-/-^* mice proliferate normally in a 3% oxygen atmosphere but senesce immediately when placed at 20% oxygen^[107]^.

Studies in human cancers show that CUX1 is overexpressed in over 70% of cancers. The Cancer Genome Atlas (TCGA) characterization of 276 human colorectal cancers ranked *CUX1* as the fifth gene on a scale showing a correlation between tumor aggressiveness and a combined score based on gene expression and somatic copy number alterations^[108]^. In glioblastomas, TCGA reveals that the chromosomal region including 7q22, where *CUX1* resides, is the most frequently and highly amplified chromosomal region^[109]^. TCGA and REMBRANDT data also show shorter survival of glioblastoma patients with high *CUX1* mRNA expression^[109] ^(reviewed in^[101]^). In smaller-scale studies, immunohistochemical analyses on breast, pancreas, and glioblastoma cancers reveal that CUX1 expression inversely correlates with relapse-free and overall survival^[101,110-112]^.

*CUX1* knockdown does not impair the clonogenic efficiency of cancer cell lines that exhibit low ROS levels but is synthetic lethal in all cancer cells that display elevated ROS levels, whether this results from an activating mutation in a *RAS *gene (Hs578T*^HRAS^*, MDA-MB-231*^KRAS^*, DLD-1*^KRAS^*, HCT116*^KRAS^*, KE37*^NRAS^*), another gene in the pathway (HT29*^BRAF^*), or an upstream receptor tyrosine kinase (HCC827*^EGFR^*)^[100,113]^. Strikingly, CUX1 knockdown does not affect the viability of A549 lung carcinoma cells which harbour an activating KRAS^G12D^ mutation^[113]^. The reason for this discrepancy is that these cells also carry an inactivating mutation in the KEAP1 tumour suppressor gene^[82,83]^. As a result, the NRF2 transcription factor accumulates in the nucleus of A549 cells and activates an antioxidant transcriptional program^[82]^.

#### Other CUT domain proteins as BER accessory factors

Structure-function analysis identified the CUT domains within CUX1 as the protein region responsible for the stimulation of OGG1, APE1, and Pol β *in vitro*^[100-102,107]^. In cells, a protein containing the CUT domains 1 and 2, together with a nuclear localization signal, was sufficient for recruitment to laser-induced DNA damage^[113]^. In cancer cells that exhibit high ROS levels, the CUT domains 1 and 2 reduced genomic DNA damage as measured by comet assay at pH 13, and comet assay at pH 10 after treatment with the Fapy DNA glycosylase (FPG)^[100-102]^. Moreover, the CUT domains 1 and 2 conferred resistance to multiple genotoxic agents, including H_2_O_2_, ionizing radiation, temozolomide, MMS, and cisplatin^[100-102,107,113]^. In addition, the CUT domains 1 and 2 can restore the capacity of *Cux1^-/-^* MEFs to proliferate in 20% oxygen^[107]^.

**Figure 3:**
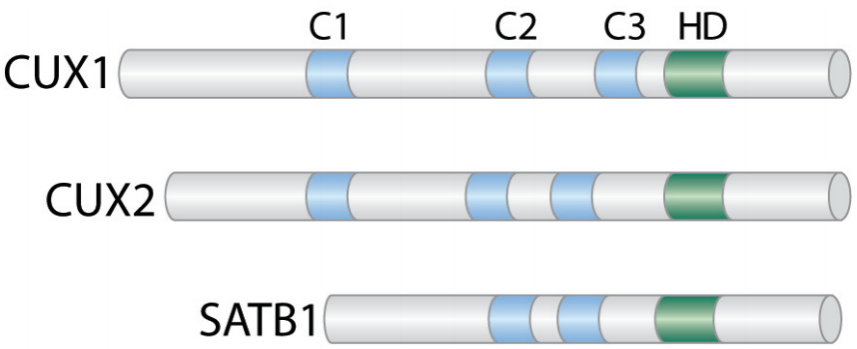
CUT Domain Proteins. Diagrammatic representation of the CUT domain proteins CUX1, CUX2, and SATB1. The evolutionarily conserved CUT domains (C) and Cut homeodomain (HD) are shown.

The CUT domains are evolutionarily conserved domains that are present in a few transcription factors in humans [Figure 3]. While CUX1 is ubiquitously expressed, CUX2 is mainly expressed in the nervous system^[114-116]^ and can also function as a female-specific transcription factor in the liver^[117]^. SATB1 is present in specific cell types such as thymocytes and basal layer cells of the epidermis where it regulates the expression of a large set of genes by organizing specific chromosome loci into small chromatin loops^[118-121]^. SATB1 has been implicated in various cancers such as breast cancers, cutaneous malignant melanoma, gastric and colorectal cancers^[122-125]^.

Not surprisingly, these other CUT domain proteins were found to function as BER accessory factors [Figure 1]. Knockdown of CUX2 or SATB1 was found to cause a delay in the repair of oxidative DNA damage and an increase in oxidized purines in genomic DNA, whereas ectopic expression of these proteins accelerated DNA repair and reduced the amount of genomic DNA lesions^[126,127]^. As for CUX1, enzymatic assays with purified proteins mapped the DNA repair activity of CUX2 and SATB1 to the CUT domains^[126,127]^. Strikingly, both CUX2 and SATB1 are overexpressed in certain cancer cells where their knockdown increases genomic DNA damage and impairs the capacity of cancer cells to proliferate^[126,127]^. The case of CUX2 is particularly remarkable. CUX2 is not expressed in the mammary gland. Yet, a genome-wide shRNA screen revealed that CUX2 is required for proliferation in several breast cancer cell lines^[128]^. The aberrant overexpression of CUX2 in breast cancer cells suggests that elevated ROS levels in cancer cells can select rare cells that express any protein able to accelerate the repair of oxidative DNA damage.

The mechanism by which CUT domains stimulate the enzymatic activity of BER enzymes is not always clear. In the case of OGG1, electrophoretic mobility shift assays (EMSA) established that CUT domains stimulate the binding of OGG1 to DNA that contains oxidized purines^[107]^. On the one hand, CUT domains can interact directly with OGG1^[107,126,127]^. On the other hand, in EMSA, CUT domains were observed to exhibit affinity to DNA that contains an oxidized purine (Ramdzan and Nepveu, unpublished observation). CUT domains were also shown to interact directly with APE1 and Pol β^[101,102]^. However, whether CUT domains accelerate the binding of these enzymes to their DNA substrates remains to be verified.

#### Other transcription factors that function as BER accessory factors

Other transcription factors and DNA binding proteins were reported to play a role in BER. YB-1 and hnRNP-U were shown to stimulate NEIL1^[129,130]^. HMGB1 was reported to stimulate the functions of the APE1 and FEN1 endonucleases^[131]^. The FACT complex was also shown to facilitate uracil removal by UDG^[132]^. *In vitro, *P53 stimulates the activity of Pol β^[133]^. These findings suggest that there may be a wide variety of accessory proteins acting to facilitate BER.

## CONSEQUENCES OF INCREASED BER ACTIVITY IN CANCER CELLS

Apart from being able to avoid senescence and continue to proliferate, the increased DNA repair capability of cancer cells that overexpress BER enzymes and accessory factors confers additional properties that have a major impact on resistance to treatment, tumour progression, and ultimately, patient survival.

### Impact of BER enzymes on resistance to treatments

There is ample evidence to demonstrate that resistance to genotoxic treatments correlates with expression and activity of several BER enzymes, notably Pol β, APE1, and FEN1 (reviewed in^[134]^). High Pol β expression confers resistance to genotoxic treatments^[135-139]^. Conversely, Pol β knockdown or inhibition sensitizes cancer cells to various treatments including temozolomide^[137,139-142]^, MMS^[143-146]^, oxaliplatin^[147]^, cisplatin and UV-radiation^[148]^. Elevated APE1 expression in cancer cells has been associated with poor response to mono-alkylating agents^[91,149-151]^, whereas various approaches to reduce APE1 activity were shown to increase cancer cell sensitivity to temozolomide^[149,152-158]^. Knockdown or inactivation of the FEN1 gene sensitized cells to gamma-radiation, MMS, temozolomide, and cisplatin^[159,160]^. The N-methylpurine DNA glycosylase (MPG) was also shown to promote resistance to mono-alkylating agents^[161,162]^. However, results also emphasized the importance of balancing glycosylase activity with that of Pol β^[137,139]^. Indeed, unless repair of the base lesion is brought to completion, removal of alkylated bases produces abasic sites and single-strand breaks that can be more toxic than the original lesions.

### Impact of BER accessory factors on resistance to treatment

As a by-product of their adaptation to high ROS levels, cancer cells that have increased their capacity to repair oxidative DNA damage through elevated expression of BER accessory factors also exhibit increased resistance to genotoxic treatments. Here, we will describe the documented effects of BER accessory factors on resistance to treatment.

There exists old literature linking RAS oncogenes with radioresistance^[163-166]^. We consider it likely that the higher resistance to ionizing radiation of some RAS-driven cancer cells is a by-product of their adaptation to elevated ROS levels through the enhanced expression of BER enzymes and accessory factors. We previously mentioned that *CUX1* knockdown did not affect the viability of cancer cell lines with low ROS levels. Strikingly, however, knockdown of *CUX1* or *SATB1* sensitized all tested cancer cell lines to ionizing radiation, whether they displayed high ROS levels or not^[101,113,127]^. In turn, ectopic expression of CUX1 or the small recombinant protein containing only two CUT domains increased the resistance to radiation^[101,113]^. In glioblastoma cells, resistance to the mono-alkylating agent temozolomide was reduced by CUX1 knockdown but increased by ectopic expression of CUX1 or the two CUT domains^[101]^. Similar results were obtained following treatment with another alkylating agent, MMS^[101]^. The standard-of-care treatment for glioblastoma patients involves a combined treatment with ionizing radiation and temozolomide. Resistance to combined treatment was reduced by *CUX1* knockdown but increased by overexpression of CUX1 or the two CUT domains^[101]^. Moreover, the resistance of cancer cells to cisplatin treatment was decreased by *CUX1* knockdown but increased by ectopic expression of the two CUT domains^[102]^. These findings are in line with the results of *in vitro* assays showing that the CUT domains stimulate the cleavage activity of APE1 as well as the deoxyribose phosphate lyase and the polymerase activities of Pol β, and the bypass of intrastrand G-crosslink by Pol β^[101,102]^.

### Contribution of elevated ROS levels and BER activity to acquired resistance and tumour progression

We previously described that the ~30,000 endogenous, daily, damaged bases in diploid human cells combined with a Pol β error rate of at least 1/1000 contributes to the intrinsic mutation process that makes our somatic cells accumulate mutations through life. This process is exacerbated in cancer cells in which increased BER activity enables them to survive despite elevated ROS levels. These cells suffer a much higher number of oxidative DNA lesions than normal cells; and assuming a similar error rate by Pol β, it is reasonable to assume that these cells exhibit a mutator phenotype and acquire point mutations at a faster rate. This notion received confirmation from a study of acquired resistance to imatinib by chronic myelogenous leukemia (CML) cells^[49]^. The authors showed that the BCR-ABL kinase causes an increase in ROS levels and oxidative DNA damage that is associated with a rise in mutation rates as measured by resistance to ouabain or imatinib^[49]^. These effects were reduced by treatment with antioxidants or expression of a BCR-ABL^Y177F^ mutant that does not elevate ROS^[49]^. ROS production induced by BCR-ABL was subsequently shown to increase chromosomal aberrations^[167]^.

The acquisition of resistance to imatinib by some BCR-ABL CML cells illustrates another consequence of elevated ROS levels and BER activity. The higher rate of mutation in these cancer cells contributes to tumour heterogeneity, a feature that constitutes a major obstacle to successful clinical treatments. Although a specific treatment can manage to kill more than 99.99% of cancer cells, if only one rare cancer cell acquires a mutation that allows its survival, selective pressure will promote the expansion of this cell clone. At the clinical level, this leads to cancer relapse.

## CONCLUDING REMARKS

Evidence from *in vitro* assays with purified proteins, tissue culture cells, and transgenic mice reveals that the efficiency of BER in mammalian cells can be modulated by the action of auxiliary factors, notably the CUT domain proteins. Although these accessory factors are not essential, they appear to protect cells against oxidative DNA damage in situations of oxidative stress. This biochemical activity is hijacked by cancer cells to avoid senescence and continue to proliferate in the presence of excessive ROS levels [Figure 2]. As a by-product of this mechanism of adaptation to elevated ROS levels, cancer cells with enhanced BER activity exhibit increased resistance to genotoxic treatments and a higher mutation rate that contributes to tumour heterogeneity. However, the acute dependence of some cancer cells on DNA repair accessory factors may have uncovered an Achilles’ heel that could be exploited in future therapeutic strategies. In the past, the realization that BER enzymes contribute to therapy resistance led to many drugs that inhibit BER enzymes being tested in the clinic with various treatment modalities. The drawback of such approaches is that BER enzymes are essential to normal cell viability, since over 30,000 base alterations per day are produced endogenously in a normal human cell^[12]^. Consequently, although inhibitors of BER enzymes increase cell killing within the tumor, they also cause severe adverse effects that considerably reduce the therapeutic window. As DNA repair accessory factors are not essential to normal cells in regular physiological situations, specific inhibitors to these proteins could be deleterious to cancer cells without causing many adverse effects, thereby increasing the therapeutic window.

## DECLARATIONS

### Copyright

© The Author(s) 2022.
